# Supplementary material for: Safety and Suitability of Infant Formula Manufactured from Extensively Hydrolyzed Whey Protein Compared to Intact Protein: A Combined Analysis of Two Randomized Controlled Studies
Source: Nutrients. 2024 Jan 12;16(2):245. doi: 10.3390/nu16020245 (PMC10821206; doi:10.3390/nu16020245)
Supplement: Supplementary file 1 [file nutrients-16-00245-s001.zip › nutrients-2783282-supplementary.pdf]

Safety and Suitability of Infant Formula Manufactured from Extensively Hydrolyzed Whey Protein Compared to Intact Protein: A Combined Analysis of Two Randomized Controlled Studies

Supplementary material

Table S1. Nutritional characteristics of the formulas.

	extensive hydrolysate		intact protein	
	eHF**	LPeHF+Syn*	iPF	LPiPF
Energy [kcal/ 100 mL]	67	67	67	67
Protein	2.3	1.9	2.2	1.89
Degree of hydrolysis	23-29%		Intact protein	Intact protein
	> 6.000 Dalton: < 0.1<br>3.500-6.000 Dalton: ~0.4<br>1.500-3.500 Dalton: ~12.7<br>< 1.500 Dalton: ~86.8		--	--
Carbohydrates [1]	10.6	10.7	12.0	11.4
Fat	5.4	5.4	4.9	5.3
GOS [g/ 100 mL]	--	0.3	--	--
L. fermentum [cfu/g] at production	--	107	--	--

Values are expressed as [g/ 100 kcal] unless otherwise indicated. All values refer to raw materials. Due to technological processes and differences in raw material, values might not exactly reflect reference values given in correspondent directive. [1] Lactose is the only carbohydrate source in all infant formulae. eHF - infant formula manufactured from extensively hydrolysed protein (2.3 g protein/100kcal), iPF – infant formula manufactured from intact protein (2.2 g protein/100kcal), LPeHF+Syn - low protein infant formula manufactured from extensively hydrolysed protein with synbiotics (1.9 g protein/100kcal), LPiPF - low protein infant formula with manufactured from intact protein (1.9 g protein/100kcal).

Table S2. Retrospective sample size calculation.

	Step 1<br>eHF vs. iPF		Step 2<br>LPeHF+Syn vs. LPiPF	
**Assumptions**	PPS	FAS	PPS	FAS
Power	80%	80%	80%	80%
Significance level	2.5%	2.5%	2.5%	2.5%
Difference between groups (g/day) [1]	−0.3	0.1	0.8	0.4
Pooled standard deviation (g/day)	6.56	6.10	6.16	6.73
Evaluable participants needed	94	62	43	63

[1] Differences were derived from average daily weight gain between 4 months and 1 month of life in respective formula groups based on the HA study [16] and BeMIM [17] study. Non-inferiority margin −3.0 g/day. eHF = infant formula manufactured from extensively hydrolyzed protein (2.3 g protein/100 kcal), FAS = full analysis set, iPF = infant formula manufactured from intact protein (2.2 g protein/100 kcal), LPeHF+Syn = low protein infant formula manufactured from extensively hydrolyzed protein with synbiotics (1.9 g protein/100 kcal), LPiPF = low protein infant formula manufactured from intact protein (1.9 g protein/100 kcal), PPS = per-protocol set.

Table S3. Baseline characteristics of study participants (FAS).

FAS (n=549)	eHF n=86	iPF n=89	LPeHF+Syn n=83	LPiPF n=88	BF n=203
	n (%)	n (%)	n (%)	n (%)	n (%)
**Maternal education**					
Basic	39 (45.4) *	7 (7.9) *	44 (53.0)	5 (5.7)	17 (8.4)
Additional	37 (43.0) *	57 (64.0) *	30 (36.1)	58 (65.9)	87 (42.9)
Tertiary	10 (11.6) *	25 (28.1)*	9 (10.8)	25 (28.4)	99 (48.8)
**Region**					
Central Europe	67 (77.9)	0 (0.0)	46 (55.4)	0 (0.0)	95 (46.8)
South-Eastern Europe	19 (22.1)	89 (100.0)	37 (44.6)	88 (100.0)	108 (53.2)
**Male sex**	39 (45.4)	45 (50.6)	39 (47.0)	43 (48.9)	104 (51.2)
**Mode of delivery (spontanous)**	52 (60.5)	58 (65.2)	42 (50.6)	58 (65.9)	150 (73.9)
**First child (yes)**	23 (26.7)	51 (57.3)	36 (43.4)	53 (60.2)	120 (59.1)
**Mother smoking before pregnancy**	38 (44.2)	44 (49.4)	34 (41.0)	46 (52.3)	63 (31.0)
**Mother smoking during pregnancy**	22 (25.6)	27 (30.3)	19 (22.9)	24 (27.3)	28 (13.8)
	Mean±SD	Mean±SD	Mean±SD	Mean±SD	Mean±SD
**Age at randomisation/ allocation (days)**	7.7±8.31	17.2±8.03	9.7±7.86	17.9±7.74	9.3±9.89
**Birth weight (g)**	3309.9±387.47	3489.1±406.92	3353.8±428.97	3380.9±382.26	3443.0±374.71
**Birth length (cm)**	50.9±1.94	51.7±1.82	51.3±2.33	51.3±1.94	51.7±1.89
**Birth head circumference (cm)**	34.9±1.31	35.3±1.12	34.8±1.36	34.9±1.12	35.2±1.29
**Age mother (years)**	30.6±4.59	30.4±5.60	31.0±5.54	30.5±5.41	30.7±4.74
**Maternal weight gain during pregnancy (kg)**	14.5±8.76	15.3±6.65	15.3±8.58	16.0±6.46 [2]	15.4±5.63
**BMI mother by before pregnancy (kg/m²)**	25.0±6.00	22.2±3.21 [7]	23.3±5.12	22.1±3.19	22.3±3.90
**Age father (years)**	33.4±5.43 [1]	[8]	34.6±7.92 [3]	[8]	34.9±6.21 [5]
**BMI father before pregnancy (kg/m²)**	26.9±6.98	26.3±3.2 [4]	26.0±3.79 [3]	26.9±4.33 [4]	26.2±4.11 [6]

Group differences (eHF vs iPF and LPeHF+Syn vs LPiPF) were tested with a Cochran-Mantel-Haenszel test adjusted for region (categorical parameters) or a vanElteren test adjusted for region (continuous data). Descriptive statistics are based on non-missing data. BF = breastfeeding; BMI = body mass index; eHF = infant formula manufactured from extensively hydrolyzed protein; iPF = infant formula manufactured from intact protein; LPeHF+Syn = low protein infant formula manufactured from extensively hydrolyzed protein with synbiotics; LPiPF = low protein infant formula manufactured from intact protein; SD = Standard deviation.[1] n=83. [2] n=84. [3] n=77. [4] n=86. [5] n=92. [6] n=197. [7] n=87. [8] Data not collected.

[*] $p < 0.05$ (2-sided).

Table S4. Average weight gain, length, and head circumference between month 1 and month 4 (FAS and PPS).

	eHF			iPF			eHF vs iPF			LPeHF+Syn			LPiPF			LPeHF+Syn vs LPiPF			BF		
	n	Mean	± SD	n	Mean	± SD	LSMean[2]	95% CI	p-value[2]	n	Mean	± SD	n	Mean	± SD	LSMean[2]	95% CI	p-value[2]	n	Mean	± SD
**FAS**																					
**Weight gain (g/day)** [1]	71	28.44	± 5.622	80	28.03	± 6.267	2.2157	[-0.807,5.238]	0.1495	69	30.60	± 7.155	84	30.47	± 6.579	0.2784	[-2.344,2.901]	0.8341	174	26.24	± 6.092
**Length gain (mm/day)**	71	1.13	± 0.217	80	0.99	± 0.170	0.1058	[0.009,0.203]	0.0325	69	1.15	± 0.195	84	1.06	± 0.171	0.0479	[-0.021,0.117]	0.1702	174	0.99	± 0.173
**Head circumference gain (mm/day)**	71	0.50	± 0.086	80	0.49	± 0.109	0.0398	[-0.007,0.087]	0.0954	69	0.54	± 0.120	84	0.49	± 0.104	0.0637	[0.024,0.103]	0.0019	174	0.49	± 0.128
**PPS**																					
**Weight gain (g/day)** [1]	39	29.03	± 5.165	42	28.45	± 6.649	0.7294	[-3.029,4.488]	0.7002	54	31.32	± 6.509	49	29.52	± 5.707	1.3921	[-1.321,4.105]	0.3111	115	26.28	± 6.149
**Length gain (mm/day)**	39	1.11	± 0.184	42	0.99	± 0.158	0.0720	[-0.027,0.171]	0.1524	54	1.17	± 0.188	49	1.06	± 0.162	0.0584	[-0.016,0.133]	0.1247	115	0.99	± 0.183
**Head circumference gain (mm/day)**	39	0.50	± 0.079	42	0.49	± 0.119	0.0323	[-0.027,0.092]	0.2821	54	0.54	± 0.124	49	0.50	± 0.108	0.0543	[0.009,0.100]	0.0192	115	0.49	± 0.127

[1] Daily gain defined as difference between month 4 and 1 divided by days in between. [2] Analysis of covariance adjusted for sex, region, baseline value at the age of 1 month. 2-sided p-values based on superiority testing at 5% significance level (not adjusted for multiple testing). LSMeans: estimated least square mean difference between formula groups (eHF – iPF, LPeHF+Syn – LPiPF), 95% CI: 95% confidence interval of estimated least square mean difference between formula groups (eHF – iPF, LPeHF+Syn – LPiPF)

Table S5. Weight-for-age and BMI-for-age z-scores – MMRM (FAS and PPS).

Weight-for-age z-score	eHF	iPF	eHF vs iPF			LPeHF+Syn	LPiPF	LPeHF+Syn vs LPiPF		
	LSMean (SEM)	LSMean (SEM)	LSMean Difference	95% CI	p-value[1]	LSMean (SEM)	LSMean (SEM)	LSMean Difference	95% CI	p-value[1]
**FAS**										
Model 1	-0.174 (0.0929)	-0.253 (0.1201)	0.079	[-0.2784;0.4354]	0.6646	-0.034 (0.0877)	0.020 (0.1184)	-0.054	[-0.3540;0.2466]	0.7244
Model 2	-0.114 (0.0788)	-0.287 (0.0985)	0.173	[-0.1032;0.4500]	0.2174	0.032 (0.0752)	-0.051 (0.0863)	0.084	[-0.1406;0.3076]	0.4630
**PPS**										
Model 1	-0.174 (0.1213)	-0.234 (0.1660)	0.060	[-0.3965;0.5165]	0.7943	0.051 (0.0973)	0.045 (0.1402)	0.006	[-0.3202;0.3320]	0.9714
Model 2	-0.111 (0.1028)	-0.084 (0.1365)	-0.027	[-0.3773;0.3232]	0.8781	0.152 (0.0880)	-0.034 (0.1051)	0.186	[-0.0656;0.4379]	0.1455

BMI-for-age z-score	eHF	iPF	eHF vs iPF			LPeHF+Syn	LPiPF	LPeHF+Syn vs LPiPF		
	LSMean (SEM)	LSMean (SEM)	LSMean Difference	95% CI	p-value[1]	LSMean (SEM)	LSMean (SEM)	LSMean Difference	95% CI	p-value[1]
**FAS**										
Model 1	-0.514 (0.1017)	-0.648 (0.1289)	0.134	[-0.2488;0.5159]	0.4916	-0.464 (0.0865)	-0.218 (0.1157)	-0.247	[-0.5403;0.0469]	0.0990
Model 2	-0.454 (0.1090)	-0.731 (0.1376)	0.277	[-0.1126;0.6662]	0.1623	-0.303 (0.1002)	-0.272 (0.1170)	-0.032	[-0.3332;0.2699]	0.8361
**PPS**										
Model 1	-0.385 (0.1322)	-0.519 (0.1755)	0.134	[-0.3495;0.6173]	0.5832	-0.344 (0.0979)	-0.245 (0.1408)	-0.099	[-0.4265;0.2293]	0.5523
Model 2	-0.327 (0.1565)	-0.386 (0.2087)	0.060	[-0.4807;0.6005]	0.8257	-0.178 (0.1296)	-0.328 (0.1562)	0.151	[-0.2177;0.5189]	0.4187

Model 1: fixed factors formula group, visit, interaction term formula group and visit, region, random factor subject.
Model 2: fixed factors formula group, visit, interaction term formula group and visit, region, random factor subject, additional covariates: maternal age at infant's birth (years), BMI at screening (kg/m^2) (mother), weight-for-age z-score (at birth) or BMI-for age z-score (at birth), maternal education (socioeconomic status), gestational age, smoking status of mother before pregnancy, smoking status of mother during pregnancy.
[1] p-value from two-sided superiority testing.
LSMean = Least squares estimation of mean derived from mixed model with repeated measurements (MMRM), SEM = estimated standard error of the mean.

Visit and z-score at birth have shown to be significantly important in explaining overall weight or BMI development, however no model showed a significant difference between formula groups over time in weight-for-age or BMI-for-age z-scores.

Table S6. Intake of study formula, other infant formula, energy containing liquids, and complementary food between month 1 and month 4 (FAS).

FAS		eHF				iPF				LPeHF+Syn				LPiPF				BF			
	Age	n	Mean	±	SD	n	Mean	±	SD	n	Mean	±	SD	n	Mean	±	SD	n	Mean	±	SD
**Formula intake**																					
Average amount (mL)/day	1 month	83	665.9	±	156.46	86	628.6	±	225.75	83	682.4	±	191.37	86	636.00	±	186.33	0			
	2 months	75	772.4	±	212.78	82	762.9	±	223.10	74	767.5	±	181.633	85	753.00	±	203.98	0			
	3 months	72	825.5	±	206.70	80	844.8	±	225.29	68	788.1	±	174.00	84	765.80	±	177.50	0			
	4 months	71	890.5	±	179.33	80	917.1	±	254.53	68	864.9	±	177.44	83	792.57	±	185.09	0			
Average energy (kcal)/day	1 month	83	446.2	±	104.83	86	421.2	±	151.25	83	457.2	±	128.22	86	426.1	±	124.84 *	0			
	2 months	75	517.5	±	142.56	82	511.1	±	149.48	74	514.2	±	121.69	85	504.5	±	136.66	0			
	3 months	72	553.1	±	138.49	80	566.0	±	150.94	68	528.1	±	116.58	84	513.1	±	118.93	0			
	4 months	71	596.7	±	120.15	80	614.5	±	170.54	68	579.5	±	118.88	83	531.0	±	124.01 *	0			
**Breastfeeding**																					
Average number of breastfeedings per day	1 month	8	4.08	±	2.730	38	2.42	±	1.840	5	2.07	±	1.256	38	2.37	±	2.432	201	8.06	±	1.595
	2 months	2	1.33	±	0.471	14	2.79	±	1.968	3	3.89	±	2.219	14	2.29	±	2.585	184	7.41	±	1.761
	3 months	1	0.67			10	2.40	±	2.066	1	5.67			7	2.57	±	2.820	178	7.02	±	1.697
	4 months	0				7	3.43	±	2.440	1	6.00			5	3.20	±	3.194	172	6.77	±	1.726
**Energy containing liquid intake**																					
Average amount (mL)/day	1 month	2	87.5	±	88.39	50	41.8	±	46.70	0				53	35.7	±	23.33	34	31.4	±	28.78
	2 months	3	83.3	±	14.43	51	41.8	±	29.22	0				54	33.4	±	23.99	34	35.1	±	22.91
	3 months	1	75.0			39	46.7	±	32.61	0				42	45.0	±	49.83	30	32.1	±	23.85
	4 months	2	137.5	±	88.39	34	28.6	±	19.21	1	75.0			39	42.6	±	29.89	23	32.9	±	28.16
Average energy (kcal)/day	1 month	2	13.9	±	12.11	50	7.0	±	11.29	0				53	5.1	±	3.29	34	4.4	±	4.03
	2 months	3	14.1	±	2.55	51	5.9	±	4.13	0				54	4.7	±	3.38	34	5.0	±	3.31
	3 months	1	16.1			39	6.6	±	4.67	0				42	6.4	±	6.99	30	4.5	±	3.33
	4 months	2	23.1	±	9.81	34	4.3	±	2.72	1	16.1			39	6.5	±	5.75	23	6.2	±	6.41
Average liquid amount (mL)/day over 4 months	Over all visits	5	61.25	±	51.615	71	24.71	±	22.338	1	18.8			69	26.3	±	23.94	58	17.2	±	15.538

For formula intake group differences in energy intake were tested with a vanElteren test adjusted for region (2-sided, continuous parameters). Statistical testing could not be performed for energy containing liquid intake due to the low sample size for data derived from the HA study.
* $p<0.05$ for LPeHF+Syn vs LPiPF.

Table S7. Intake of study formula, other infant formula, energy containing liquids, and complementary food between month 1 and month 4 (PPS).

PPS		eHF				iPF				LPeHF+Syn				LPiPF				BF			
	Age	n	Mean	±	SD	n	Mean	±	SD	n	Mean	±	SD	n	Mean	±	SD	n	Mean	±	SD
**Formula intake**																					
Average amount (mL)/day	1 month	39	677.9	±	157.05	42	698.1	±	200.36	54	695.8	±	172.56	49	666.6	±	169.32	0			
	2 months	39	767.2	±	155.56	42	812.5	±	193.84	54	784.3	±	181.45	49	764.7	±	196.95	0			
	3 months	39	820.6	±	222.01	42	887.0	±	211.22	54	801.9	±	170.75	49	794.3	±	157.83	0			

	4 months	39	879.4	±	184.60	42	953.1	±	215.87	54	889.9	±	152.85	49	824.8	±	176.92		0			
Average energy (kcal)/day	1 month	39	454.2	±	105.23	42	467.8	±	134.24	54	466.2	±	115.61	49	446.6	±	113.45		0			
	2 months	39	514.0	±	104.23	42	544.4	±	129.87	54	525.5	±	121.57	49	512.4	±	131.95		0			
	3 months	39	549.8	±	148.75	42	594.3	±	141.52	54	537.2	±	114.40	49	532.2	±	105.75		0			
	4 months	39	589.2	±	123.68	42	638.6	±	144.63	54	596.3	±	102.41	49	552.6	±	118.54	*	0			
**Breastfeeding**																						
Average number of breastfeedings per day	1 month	0				11	1.00	±	0.000	0				17	1.00	±	0.000		115	8.03	±	1.594
	2 months	0				2	1.00	±	0.000	0				4	1.00	±	0.000		115	7.54	±	1.567
	3 months	0				0				0				1	1.00				115	7.16	±	1.641
	4 months	0				0				0				2	1.00	±	0.000		115	7.03	±	1.500
**Energy containing liquid intake**																						
Average amount (mL)/day	1 month	1	25.0			27	37.1	±	30.04	0				26	35.3	±	21.0		12	28.3	±	27.36
	2 months	1	75.0			22	34.0	±	15.84	0				29	25.6	±	13.91		12	19.6	±	9.93
	3 months	0				18	46.7	±	26.81	0				26	33.9	±	26.77		11	24.7	±	19.38
	4 months	0				15	23.8	±	14.43	1	75.00			22	33.0	±	21.09		9	22.4	±	13.15
Average energy (kcal)/day	1 month	1	5.4			27	5.3	±	4.43	0				26	5.0	±	3.03		12	4.00	±	3.83
	2 months	1	16.1			22	4.8	±	2.33	0				29	3.7	±	2.01		12	2.8	±	1.41
	3 months	0				18	6.7	±	3.98	0				26	4.8	±	3.79		11	3.5	±	2.71
	4 months	0				15	3.5	±	1.97	1	16.1			22	4.9	±	3.01		9	4.5	±	3.00
Average liquid amount (mL)/day over four months	Over all visits	1	25.0			35	21.1	±	14.13	1	18.8			40	20.4	±	12.40		23	11.4	±	10.36

For formula intake group differences in energy intake were tested with a vanElteren test adjusted for region (2-sided, continuous parameters). Statistical testing could not be performed for energy containing liquid intake due to the low sample size for data derived from the HA study.

* $p<0.05$ for LPeHF+Syn vs LPiPF.

Table S8. Adverse events (FAS).

	eHF			iPF			LPeHF+Syn			LPiPF			BF		
	**n′**	**N**	**(%)**	**n′**	**N**	**(%)**	**n′**	**N**	**(%)**	**n′**	**N**	**(%)**	**n′**	**N**	**(%)**
**All**	28	20	(23.26)	36	23	(25.84)	26	19	(22.89)	20	16	(18.18)	61	52	(25.62)
**Related AEs**	5	5	(5.81)	1	1	(1.12)	7	6	(7.23)	-	-	-	5	5	(2.46)
**Serious AEs**	4	3	(3.49)	2	2	(2.25)	5	3	(3.61)	6	6	(6.82)	11	10	(4.93)
**AEs by SOC**															
Gastrointestinal disorders	5	4	(4.65)	6	6	(6.74)	1	1	(1.20)	1	1	(1.14)	3	3	(1.48)
General disorders and administration site conditions	9	7	(8.14)	0	0		5	5	(6.02)	1	1	(1.14)	6	6	(2.96)
Infections and infestations	5	5	(5.81)	24	16	(17.98)	7	6	(7.23)	17	14	(15.91)	29	24	(11.82)
Metabolism and nutrition disorders*	3	3	(3.49)	-	-	-	5	5	(6.02)	-	-	-	5	5	(2.46)
Skin and subcutaneous tissue disorders	5	4	(4.65)	6	6	(6.74)	3	2	(2.41)	1	1	(1.14)	12	12	(5.91)
Respiratory, thoracic and mediastinal disorders	-	-	-	-	-	-	-	-	-	-	-	-	4	4	(1.97)

Number of events (e), number of participants with events (N) and percentage of participants with respective adverse events are indicated. Only adverse events reported by at least 2 participants in any group are listed in the by SOC display. The SOC total includes all AEs irrespective of frequency i.e., the number of events may not sum up to the SOC total number of AEs.
- indicates that no respective AE was documented. * The PT "overweight" was reported in 2 (2.33%) of eHF and in 5 (6.02%) of LPeHF+Syn infants. The PT "overweight" was not reported in iPF LPiPF or BF infants. AE = adverse event; SAE = serious adverse event; PT = preferred term; SOC = system organ class.

Table S9. Plasma amino acid profile at month 4 (FAS)**.**

FAS	Standard protein group				Low protein group					
	eHF		iPF		LPeHF+Syn		LPiPF		BF	
N	66		78		58		83		166	
Alanine	496.0	385.0, 641.0	382.0	323.0, 468.0	517.0	410.0, 590.0	360.0	301.0, 411.0	364.5	313.0, 429.0
Arginine	91.2	72.2, 112.0	92.9	75.1, 104.0	114.0	89.9, 140.0	82.4	68.6, 96.6	87.7	74.4, 102.0
Aspargic acid	15.0	12.2, 17.5	12.8	11.6, 14.5	11.9	10.3, 14.0	11.7	10.1, 13.0	11.5[b]	10.1, 13.6
Aspargine	70.5[a]	57.9, 87.7	55.8[g]	48.6, 62.6	71.9	56.7, 79.3	49.0	42.0, 57.4	46.0	38.8, 56.2
Citrulline	23.3	20.7, 29.0	22.2	18.6, 25.6	25.2	19.6, 32.8	21.2	17.4, 24.7	15.2	11.6, 19.5
Cysteine	21.9	11.8, 34.7	1.2[e]	0.4, 2.0	25.6	20.5, 34.6	1.7[d]	0.6, 3.3	5.7[c]	1.0, 15.3
Glutamine	671.5	567.0, 813.0	559.0	482.0, 629.0	751.5	649.0, 876.0	558.5[f]	477.0, 611.0	662.0	558.0, 751.0
Glutamine acid	129.0[a]	106.0, 154.0	139.5	118.0, 170.0	119.5	99.8, 148.0	142.0[f]	125.0, 169.0	152.5	124.0, 184.0
Glycine	223.5	175.0, 264.0	177.0	160.0, 196.0	243.5	192.0, 274.0	178.5[f]	156.0, 200.0	179.0	157.0, 213.0
Histidine	106.0	93.1, 128.0	88.8[g]	80.8, 99.9	96.7	85.6, 111.0	85.1	73.1, 94.5	94.5	80.0, 107.0
Isoleucine	121.5	91.3, 154.0	79.6	68.8, 89.1	94.9	77.4, 109.0	68.6	53.6, 83.2	64.0	51.4, 74.8
Leucine	163.0	131.0, 204.0	143.5	118.0, 159.0	149.5	122.0, 183.0	125.0[h]	99.0, 145.0	121.0	94.5, 135.0
Lysine	313.0	232.0, 371.0	190.5	167.0, 220.0	243.0	201.0, 274.0	191.0[f]	156.0, 221.0	166.0	134.0, 197.0
Methionine	53.2[i]	39.3, 61.7	34.6[g]	30.1, 39.1	34.0	28.5, 38.6	30.3	25.1, 35.8	24.8	20.3, 30.2
Ornithine	85.6	77.9, 99.2	87.4	75.1, 100.0	98.2	79.5, 119.0	80.1	65.7, 99.9	92.4	74.9, 111.0
Phenylalanine	52.5	42.8, 61.9	59.5	51.8, 64.6	71.5	59.1, 80.1	64.9	54.7, 76.8	48.2	41.4, 57.3
Proline	302.0	237.0, 346.0	268.0	223.0, 321.0	231.0	197.0, 249.0	212.5[f]	182.0, 252.0	268.5	223.0, 316.0
Serine	178.5	145.0, 218.0	133.5	121.0, 150.0	167.0	150.0, 198.0	135.0	121.0, 158.0	161.5	139.0, 187.0
Threonine	325.0[a]	247.0, 384.0	184.0	160.0, 231.0	258.0	209.0, 300.0	141.0	114.0, 167.0	140.0	115.0, 166.0
Tryptophane	95.1	65.6, 125.0	66.2	58.9, 73.7	105.0	87.4, 122.0	70.2	61.2, 80.8	65.8	55.9, 80.6
Tyrosine	100.5	83.3, 123.0	84.6	73.5, 100.0	100.5	84.2, 129.0	91.5[f]	77.6, 105.0	80.9	66.1, 98.0
Valine	266.0	219.0, 307.0	232.0	212.0, 259.0	193.0	168.0, 214.0	203.0[h]	168.0, 228.0	176.0	150.0, 211.0

Depicted are medians and upper and lower quartile (µmol/L). Differences between eHF vs iPF and between LPeHF+Syn vs LPiPF $P<0.05$ (2-sided, vanElteren test adjusted for region) for all aminoacids except for eHF vs iPF: Glutamine acid, Ornithine, Phenylalanine and LPeHF+Syn vs LPiPF: Aspargic acid, Proline, Valine. [a] n=65, [b] n=165, [c] n=125, [d] n=52, [e] n=43, [f] n=82, [g] n=77, [h] n=81, [i] n=64.

Table S10. Stool frequency, color and consistency (FAS).

FAS			Standard protein group					Low protein group						
			eHF		iPF		p value¹	LPeHF+Syn		LPiPF		p value¹	BF	
	Age	Category	n	(%)	n	(%)	eHF vs. iPF	n	(%)	n	(%)	LPeHF+Syn vs. LPiPF	n	(%)
Total number of stools (categorized)	1 month	1: <1/day	122	47.84	32	12.31		88	35.92	52	21.40		58	9.68
		2: 1-3/day	99	38.82	152	58.46		115	46.94	171	70.37		175	29.22
		3: 4-6/day	28	10.98	63	24.23		38	15.51	19	7.82		278	46.41
		4: 7-10/day	6	2.35	13	5.00		4	1.63	1	0.41		88	14.69
		5: >10/day	0	0.00	0	0.00	0.0005	0	0.00	0	0.00	0.3959	0	0.00
		**Total**	255	100.00	260	100.00		245	100.00	243	100.00		599	100.00
	2 months	1: <1/day	107	47.56	51	21.07		144	64.86	62	26.72		130	24.39
		2: 1-3/day	94	41.78	165	68.18		71	31.98	159	68.53		216	40.53
		3: 4-6/day	16	7.11	23	9.50		7	3.15	11	4.74		155	29.08
		4: 7-10/day	8	3.56	3	1.24		0	0.00	0	0.00		31	5.82
		5: >10/day	0	0.00	0	0.00	0.4211	0	0.00	0	0.00	<.0001	1	0.19
		**Total**	225	100.00	242	100.00		222	100.00	232	100.00		533	100.00
	3 months	1: <1/day	116	53.70	50	21.01		150	73.17	72	31.44		183	35.47
		2: 1-3/day	88	40.74	165	69.33		49	23.90	155	67.69		214	41.47
		3: 4-6/day	9	4.17	23	9.66		6	2.93	2	0.87		100	19.38
		4: 7-10/day	3	1.39	0	0.00		0	0.00	0	0.00		19	3.68
		5: >10/day	0	0.00	0	0.00	<.0001	0	0.00	0	0.00	<.0001	0	0.00
		**Total**	216	100.00	238	100.00		205	100.00	229	100.00		516	100.00
	4 months	1: <1/day	110	51.64	51	21.70		132	65.67	74	31.49		178	34.70
		2: 1-3/day	83	38.97	169	71.91		68	33.83	157	66.81		263	51.27
		3: 4-6/day	16	7.51	14	5.96		1	0.50	4	1.70		65	12.67
		4: 7-10/day	4	1.88	1	0.43		0	0.00	0	0.00		7	1.36
		5: >10/day	0	0.00	0	0.00	0.0003	0	0.00	0	0.00	<.0001	0	0.00
		**Total**	213	100.00	235	100.00		201	100.00	235	100.00		513	100.00
Dominant consistency (3 categories)	1 month	1: watery	22	9.44	1	0.39		17	7.62	2	0.82		124	21.20
		2: hard	0	0.00	10	3.92		0	0.00	18	7.41		1	0.17
		3: others	211	90.56	244	95.69	0.4393	206	92.38	223	91.77	0.9466	460	78.63
		**Total**	233	100.00	255	100.00		223	100.00	243	100.00		585	100.00
	2 months	1: watery	20	9.80	4	1.67		10	5.24	2	0.87		154	31.36
		2: hard	1	0.49	2	0.83		0	0.00	8	3.48		0	0.00
		3: others	183	89.71	234	97.50	0.8003	181	94.76	220	95.65	0.6707	337	68.64
		**Total**	204	100.00	240	100.00		191	100.00	230	100.00		491	100.00
	3 months	1: watery	28	14.58	7	2.97		16	9.64	5	2.25		104	22.76
		2: hard	0	0.00	2	0.85		0	0.00	5	2.25		0	0.00
		3: others	164	85.42	227	96.19	0.6254	150	90.36	212	95.50	0.0192	353	77.24

		**Total**	192	100.00	236	100.00		166	100.00	222	100.00		457	100.00
	4 months	1: watery	25	13.16	9	3.86		18	10.59	8	3.46		103	22.29
		2: hard	0	0.00	2	0.86		0	0.00	9	3.90		0	0.00
		3: others	165	86.84	222	95.28	0.1377	152	89.41	214	92.64	0.3518	359	77.71
		**Total**	190	100.00	233	100.00		170	100.00	231	100.00		462	100.00
Dominant color (3 categories)	1 month	1: normal	198	84.62	252	98.05		160	71.75	232	95.08		564	96.41
		2: green	36	15.38	5	1.95		62	27.80	12	4.92		21	3.59
		3: black/grey	0	0.00	0	0.00	0.0382	1	0.45	0	0.00	0.0002	0	0.00
		**Total**	234	100.00	257	100.00		223	100.00	244	100.00		585	100.00
	2 months	1: normal	157	76.96	233	97.08		104	54.45	209	92.48		442	90.02
		2: green	46	22.55	6	2.50		87	45.55	17	7.52		49	9.98
		3: black/grey	1	0.49	1	0.42	0.1040	0	0.00	0	0.00	<.0001	0	0.00
		**Total**	204	100.00	240	100.00		191	100.00	226	100.00		491	100.00
	3 months	1: normal	144	75.00	212	91.38		64	38.55	211	95.91		415	91.21
		2: green	42	21.88	18	7.76		100	60.24	9	4.09		37	8.13
		3: black/grey	6	3.13	2	0.86	0.3744	2	1.20	0	0.00	<.0001	3	0.66
		**Total**	192	100.00	232	100.00		166	100.00	220	100.00		455	100.00
	4 months	1: normal	123	64.74	228	97.44		66	38.82	212	93.81		425	92.79
		2: green	65	34.21	6	2.56		102	60.00	11	4.87		32	6.99
		3: black/grey	2	1.05	0	0.00	0.1545	2	1.18	3	1.33	<.0001	1	0.22
		**Total**	190	100.00	234	100.00		170	100.00	226	100.00		458	100.00

[1] two-sided p-value derived from Cochran-Mantel-Haenszel test adjusted for region.

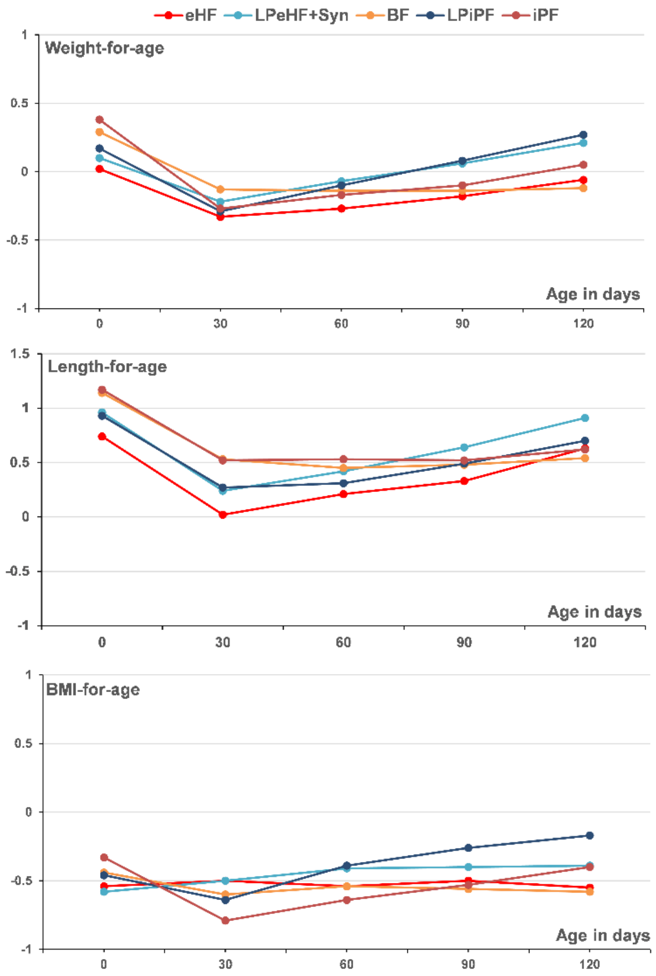

Figure S1. Anthropometric measurements (weight-for-age, length-for-age, and BMI-for-age) expressed as z scores (growth standards of the WHO) (FAS). z scores within -1 to 1 indicate an age-appropriate development. BMI = body mass index, WHO = World Health Organization.

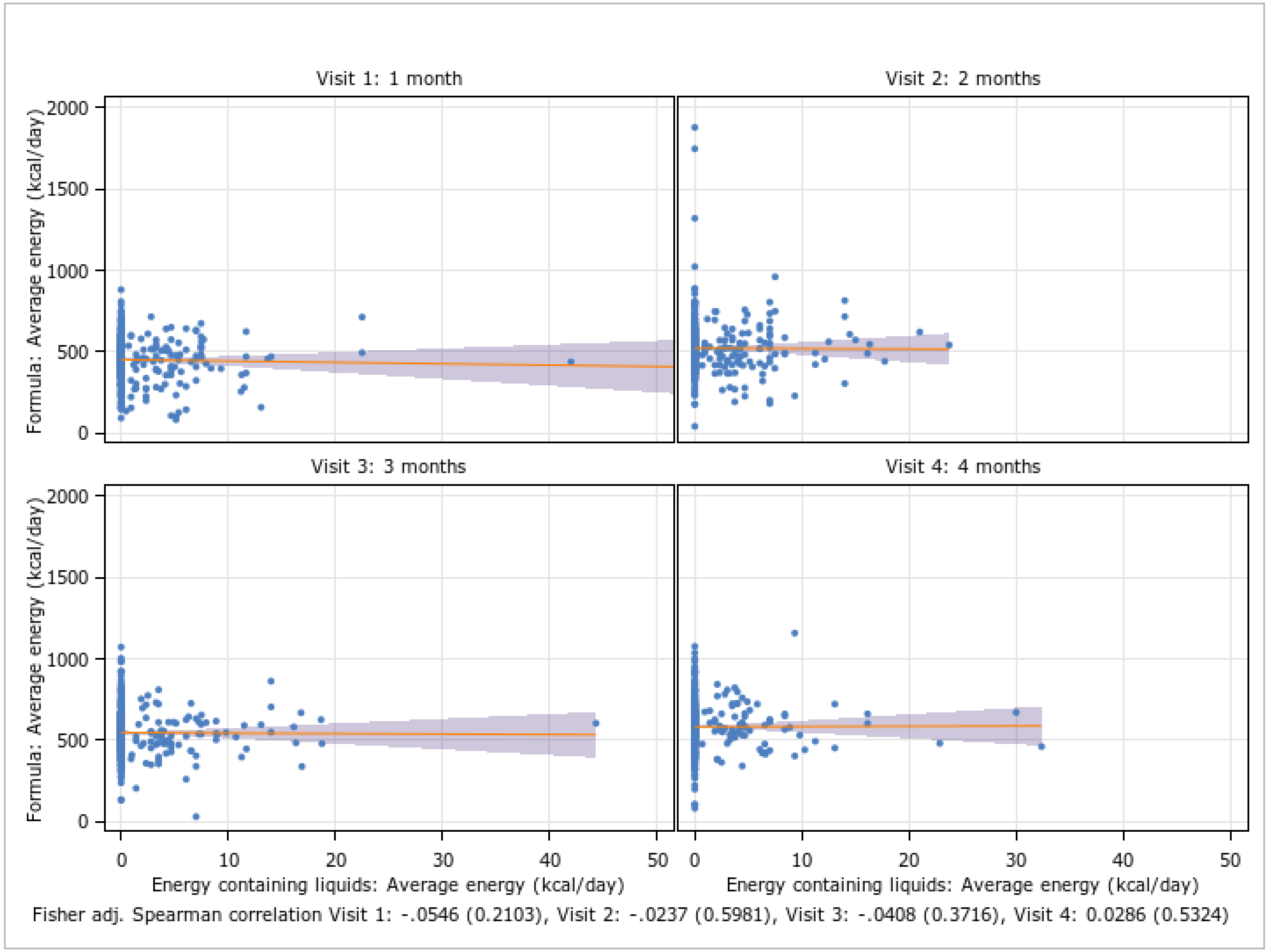

Figure S2. Scatterplots to correlate the impact of average study formula intake/day at 1, 2, 3, and 4 month(s) of life on average energy intake from liquids (FAS).

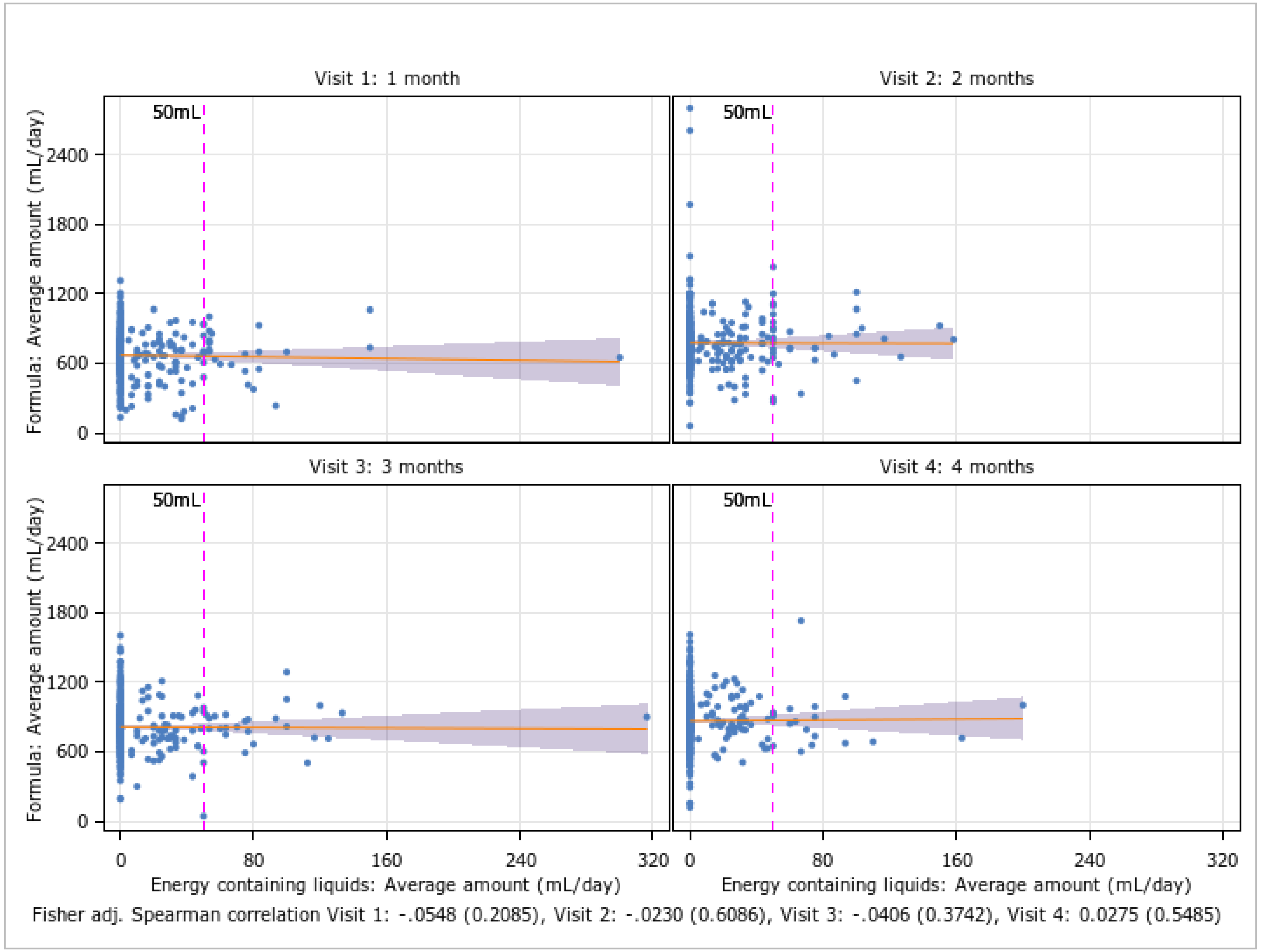

Figure S3. Scatterplots to correlate the impact of average amount of study formula intake/day at 1, 2, 3, and 4 month(s) of life on average amount of energy containing liquid intake (FAS).

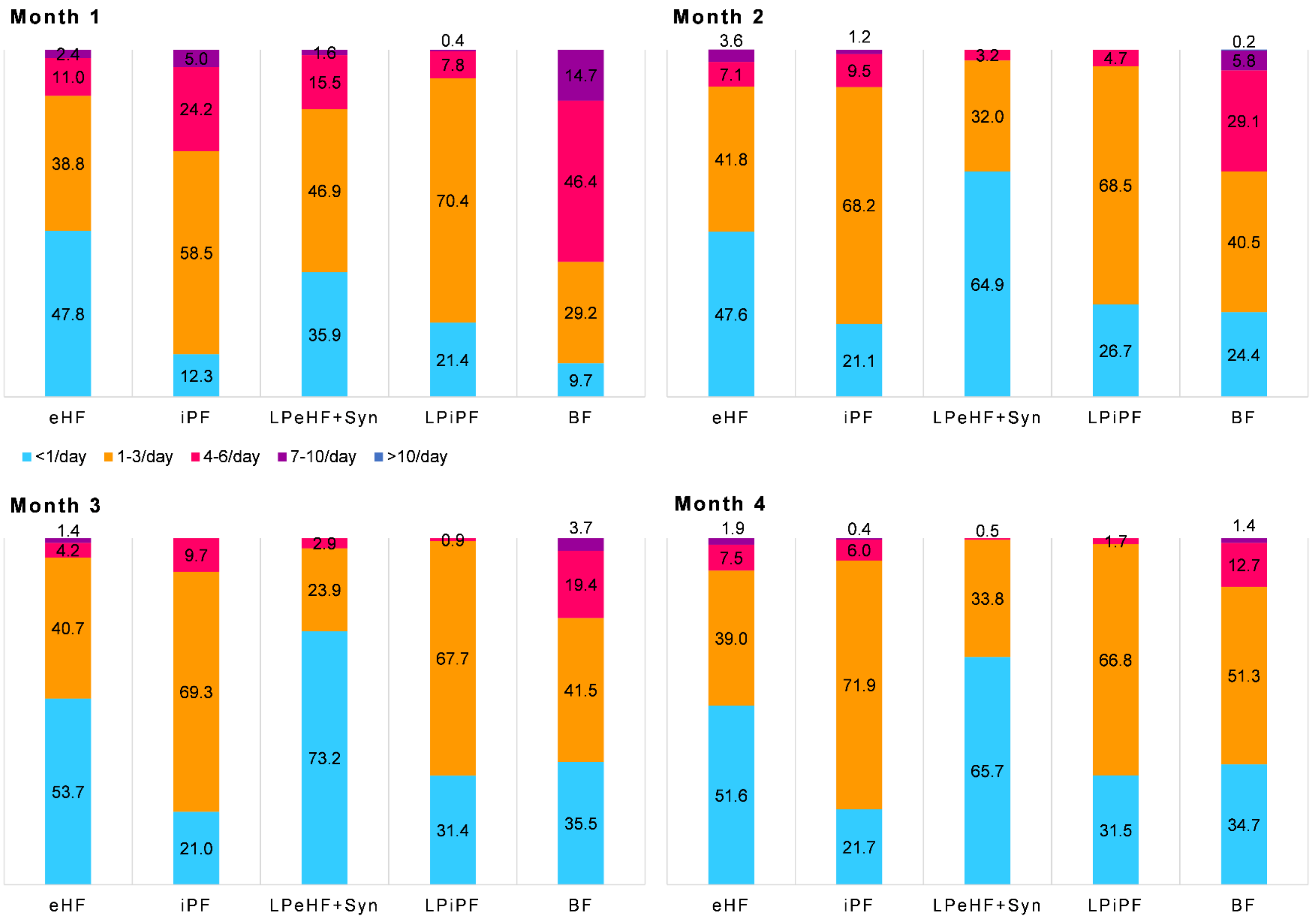

Figure S4. Stool frequency at 1, 2, 3, and 4 month(s) of life (FAS).
Values depicted represent percent of infants in the respective formula groups with the indicated number of stools/day. Significant differences between eHF vs iPF: at 1, 3, and 4 months; LPeHF+Syn vs LPiPF: at 2, 3, and 4 months.
